# A rodent model for *Dirofilaria immitis*, canine heartworm: parasite growth, development, and drug sensitivity in NSG mice

**DOI:** 10.1038/s41598-023-27537-z

**Published:** 2023-01-18

**Authors:** Jessica A. Hess, Mark L. Eberhard, Marcelo Segura-Lepe, Kathrin Grundner-Culemann, Barbara Kracher, Jeffrey Shryock, John Harrington, David Abraham

**Affiliations:** 1 Department of Microbiology and Immunology, Sidney Kimmel Medical College, Thomas Jefferson University, Philadelphia, PA USA; 2 Social Circle, GA 30025 USA; 3 Evotec, Anna-Sigmund-Straße 5, 82061 Neuried, Germany; 4 Boehringer Ingelheim Animal Health USA Inc., 6498 Jade Road, Fulton, MO USA; 5 Boehringer Ingelheim Animal Health USA Inc., 1730 Olympic Dr, Athens, GA USA

**Keywords:** Parasitic infection, Proteomics, Pharmacology

## Abstract

Heartworm disease, caused by *Dirofilaria immitis,* remains a significant threat to canines and felines. The development of parasites resistant to macrocyclic lactones (ML) has created a significant challenge to the control of the infection. The goal of this study was to determine if mice lacking a functional immune response would be susceptible to *D. immitis*. Immunodeficient NSG mice were susceptible to the infection*,* sustaining parasites for at least 15 weeks, with infective third-stage larvae molting and developing into the late fourth-stage larvae. Proteomic analysis of host responses to the infection revealed a complex pattern of changes after infection, with at least some of the responses directed at reducing immune control mechanisms that remain in NSG mice. NSG mice were infected with isolates of *D. immitis* that were either susceptible or resistant to MLs, as a population. The susceptible isolate was killed by ivermectin whereas the resistant isolate had improved survivability, while both isolates were affected by moxidectin. It was concluded that *D. immitis* survives in NSG mice for at least 15 weeks. NSG mice provide an ideal model for monitoring host responses to the infection and for testing parasites in vivo for susceptibility to direct chemotherapeutic activity of new agents.

## Introduction

Heartworm disease is caused by the presence of adult filarial nematodes, *Dirofilaria immitis,* in the pulmonary vascular system of canids. Infections with this parasite are found throughout the tropical and temperate regions of the world, with prevalence increasing in many areas. Mosquitoes transmit *D. immitis* after the female insects acquire microfilariae (mf) from infected dogs during a blood meal. Mf develop into infective third-stage larvae (L3) in mosquitoes, emerge from the proboscis of the insect, and enter a new mammalian host via the wound inflicted during feeding. The L3 molt to fourth-stage larvae (L4) within several days, migrate via a yet unknown path, and ultimately molt to pre-adults that enter the circulatory system where mf are produced by female adult worms thereby completing the lifecycle. This process of migration, molting and sexual reproduction takes approximately 6–7 months to complete. The development and host–pathogen interactions of *D. immitis* during the pre-patent period, from initial parasite entry through migration and molting to adults, have been very difficult to study, resulting in many unanswered questions. However, the prepatent period provides a critical window of opportunity to prevent heartworm disease through appropriate use of macrocyclic lactones (ML). Currently, ML are the only class of drugs approved to prevent development of heartworm disease. Prevention is accomplished through neutralization of the larval stages in the mammalian host such that presence of adults is precluded^1–3^. Larvae are sensitive to ML-mediated neutralization for approximately 60 days post entry into the mammalian host, after which a precipitous decrease in efficacy of ML is observed^4^. Finally, resistance to MLs, has been documented in *D. immitis*, creating additional challenges to preventing heartworm disease^2,5,6^.

The typical host for *D. immitis* is the domestic dog, with other canines susceptible to the infection, potentially supporting the complete life cycle. Other hosts, including felines, have also been described which are capable of supporting non-patent *D. immitis* infections^1,3^. Detailed descriptions have been made of the developmental stages of *D. immitis* in dogs from the L3 to the adult stages^7–9^. Attempts have been made to identify a small animal host that would provide a suitable model for laboratory investigations of heartworm disease. *D. immitis* L3 injected into various strains of mice were rapidly eliminated^10^. As an alternative approach for the study of the early stages of *D. immitis* infection in dogs and in mice, diffusion chambers constructed from Lucite rings to which porous membranes are attached, were utilized. L3 injected into diffusion chambers are too large to escape through the membrane pores yet host cells and humoral factors can freely enter. *D. immitis* L3 inserted into diffusion chambers and then implanted subcutaneously into mice or dogs were shown to survive and grow at equal rates for at least three weeks, although the rates of development were reduced from that described in tissues of dogs^8,11^.

NOD.Cg-*Prkdc*^*scid*^*Il2rg*^*tm1Wjl*^/SzJ (NSG) mice have profound defects in both innate and adaptive and immune responses^12^ including: (1) loss of both T and B cell function^13^, (2) macrophages, NK cells and dendritic cells are found in NSG mice but are functionally compromised, (3) defects in the complement cascade^14,15^, (4) eosinophils are found in the bone marrow of NSG mice but not in the peripheral blood^16^ and (5) monocytes and neutrophils are present in NSG mice although their functional capacity is unknown^15^. It was hypothesized that the absence of a functional innate and adaptive immune response would enhance the susceptibility of these mice to infection with nematode parasites, as opposed to the immunocompetent mice which were resistant to the infections. This hypothesis was validated based on the observation that NSG mice were susceptible to the complete life cycle of *Strongyloides stercoralis* while immunocompetent mice were resistant. All stages normally found in humans were present in NSG mice and hyperinfection developed when mice were treated with a glucocorticoid, as has been reported in humans. Furthermore, the *S. stercoralis*/NSG mouse model was successful at identifying a new therapeutic agent to control hyperinfection with *S. stercoralis*^16^. NSG mice have also proven to be sufficient hosts for filarial worms. *Loa loa* L3 injected into NSG mice develop into adult males and females that produce mf. *L. loa* survived in NSG mice for at least 5 months^17^. Finally, NSG and humanized-NSG mice were infected with the L3 of *Onchocerca volvulus.* Given the long period of time required for adult worms to develop in susceptible hosts and the size of the adult worms, it was anticipated that complete life cycle of *O. volvulus* would not be possible in NSG mice. However, parasites did survive for at least 3 months, increased in length four-fold and developed into advanced L4. Infected humanized-NSG mice were used to identify *O. volvulus-*derived proteins as potential biomarkers in serum and urine collected from the infected mice^18^.

The goals of the present study were to assess the survival, growth and development of *D. immitis* L3 injected into NSG mice. Proteomic analyses were performed to identify changes in the NSG mice during infection with *D. immitis*. Finally, the *D. immitis*/NSG model was tested to determine its utility as a tool for analyzing drug efficacy against susceptible and resistant isolates of *D. immitis.*

## Results

### NSG mice are susceptible to infection with *D. immitis* L3

C57BL/6J, NOD, SCID, and NOD/SCID and NSG mice were infected by subcutaneous injection of 50 *D. immitis* L3. Six weeks post-infection the mice were necropsied, and worms collected from the tissues. NSG mice had an average worm-recovery of 29% ± 10, while no worms were recovered from the C57BL/6J, NOD, SCID, or NOD/SCID mice. Worms were only recovered from the skin and muscle and not from the peritoneal cavity or the visceral tissues. NSG mice were infected with 25, 50, or 100 L3 and after 6 weeks the mice were necropsied, and worms collected from the tissues. There were no significant differences in the percentage of parasites recovered based on the infection dose. Mice infected with 25 L3 had a mean recovery rate of 16% ± 7, mice infected with 50 L3 had a mean recovery rate of 22% ± 7 and mice infected with 100 L3 has a mean recovery rate of 21% ± 8.

### Growth of *D. immitis* in NSG mice

**Figure 1:**
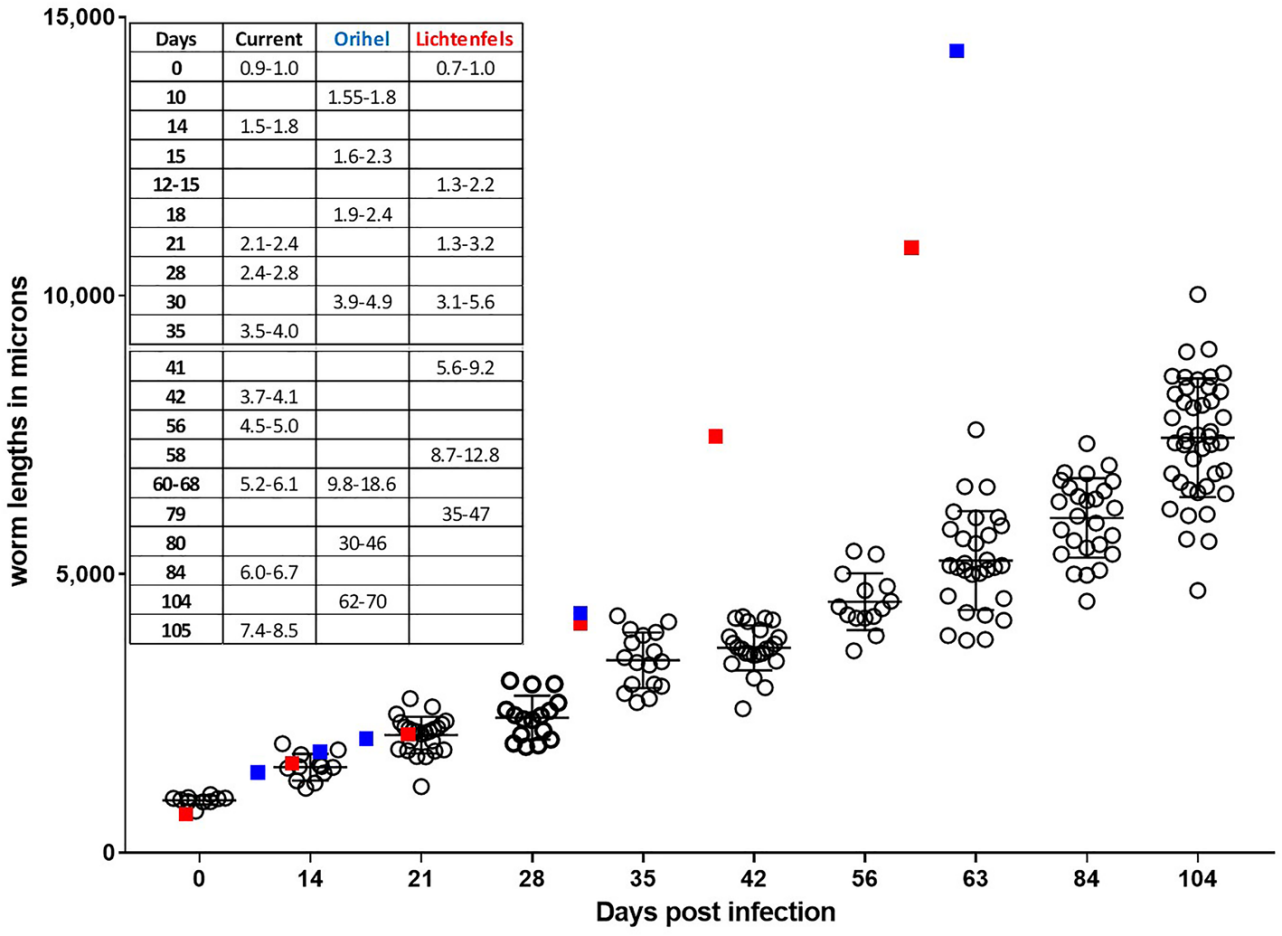
Growth curve for larva of *Dirofilaria immitis* injected subcutaneously into NSG mice. Fifty L3 were injected, and parasites were recovered at various time points over 105 days. Table insert presents the range of sizes of parasites, for comparative purposes, recovered in the present study (current) and from two studies in which parasites were recovered from dogs, Orihel^9^ and Lichtenfels^8^. Blue squares denote approximate size of worms recovered from the Orihel study and red squares approximate size of worms recovered from the Lichtenfels study.

NSG mice, infected with 50 L3, were necropsied over a 15-week time period and the recovered worms measured. L3’s measured on day 0 had mean length of 939 ± 79 µm. By 105 days post-infection the mean length was 7488 ± 1065 µm with a maximum length of 10,021 µm recorded (Fig. 1). Analysis of the lengths of the recovered worms suggested that the parasite growth rate was linear in the NSG mice over the 15 week infection period with R^2^ coefficient of determination = 0.9797. For the purposes of comparison, the lengths of *D. immitis* recovered from dogs at different time points from two previous studies (Orihel^9^ and Lichtenfels et al.^8^ were added to Fig. 1). During the first 30–35 days of infection, worms recovered from NSG mice and from dogs apparently develop at the same rate achieving equivalent lengths. After this time period*, D. immitis* in dogs developed at a faster rate attaining greater lengths than worms recovered from the mice (Fig. 1).

### Morphological development of *D. immitis* in NSG mice

A number of larvae recovered from mice at 14 days were observed to be in the process of molting. This molt at 9–14 days constitutes the third overall molt and the first molt in the vertebrate host. No molting larvae were observed between 8 and 12 weeks in the present study.

The internal morphology of developing larvae was similar to what has been described for larvae recovered from dogs but benchmark changes appeared at later time points. In 2-week-old larvae, the male and female genital primordia were still small, composed of only a few cells, but more visible than in the L3. By week 3, the female ovijector was attached to the body wall while male testis had developed the characteristic posteriorly directed or C-shaped attitude. Small spicule pads could be seen in male larvae lying on either side of the rectum just anterior to the anal opening. The ovijector remained in the esophageal region until week 9, when due to body growth, it was now located posterior to the esophageal-intestinal junction. During weeks 3–15, the reproductive systems in both male and female larvae continued to grow such that in female larvae, the paired uteri and ovaries grew in length and extended well into the posterior body. In male larvae, the testis continued its posteriorly directed growth and by approximately 8 weeks, the posterior end of the reproductive tube reached the posterior end of the worm and had formed, in conjunction with the intestine, the combined cloaca. By week 12, both male and female reproductive systems showed considerable looping in the body cavity. The spicule pads grew from approximately 20 µm in length at 3 weeks to a maximum of about 125 µm by week 12.

**Figure 2:**
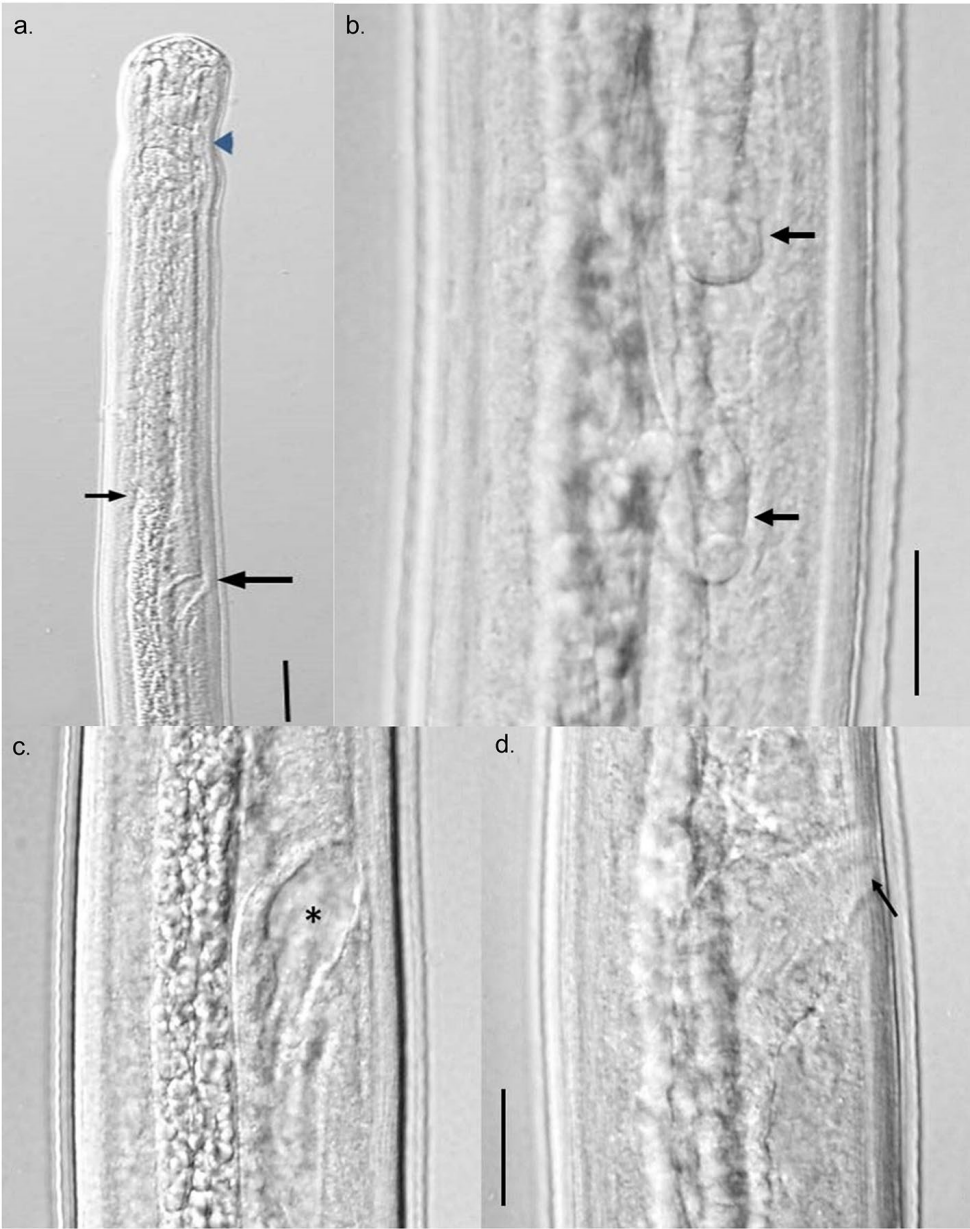
Differential Interference Contrast microscopy image of female larva recovered from NSG mice at 15 weeks post infection. (**a**) Anterior end showing nerve ring (arrow head), junction of esophagus and intestine (small arrow), and ovijector (large arrow). Scale bar 50 µm. (**c**,**d**) Composite image of ovijector, (**c**) hollow cavity (asterisk) and posteriorly directed vagina. Scale bar 20 µm. (**d**) Narrow, muscular vulva (arrow) attached to body wall. (**b**) Paired coiled ovaries in the posterior quarter of the body (arrows). Scale bar 20 µm.

**Figure 3:**
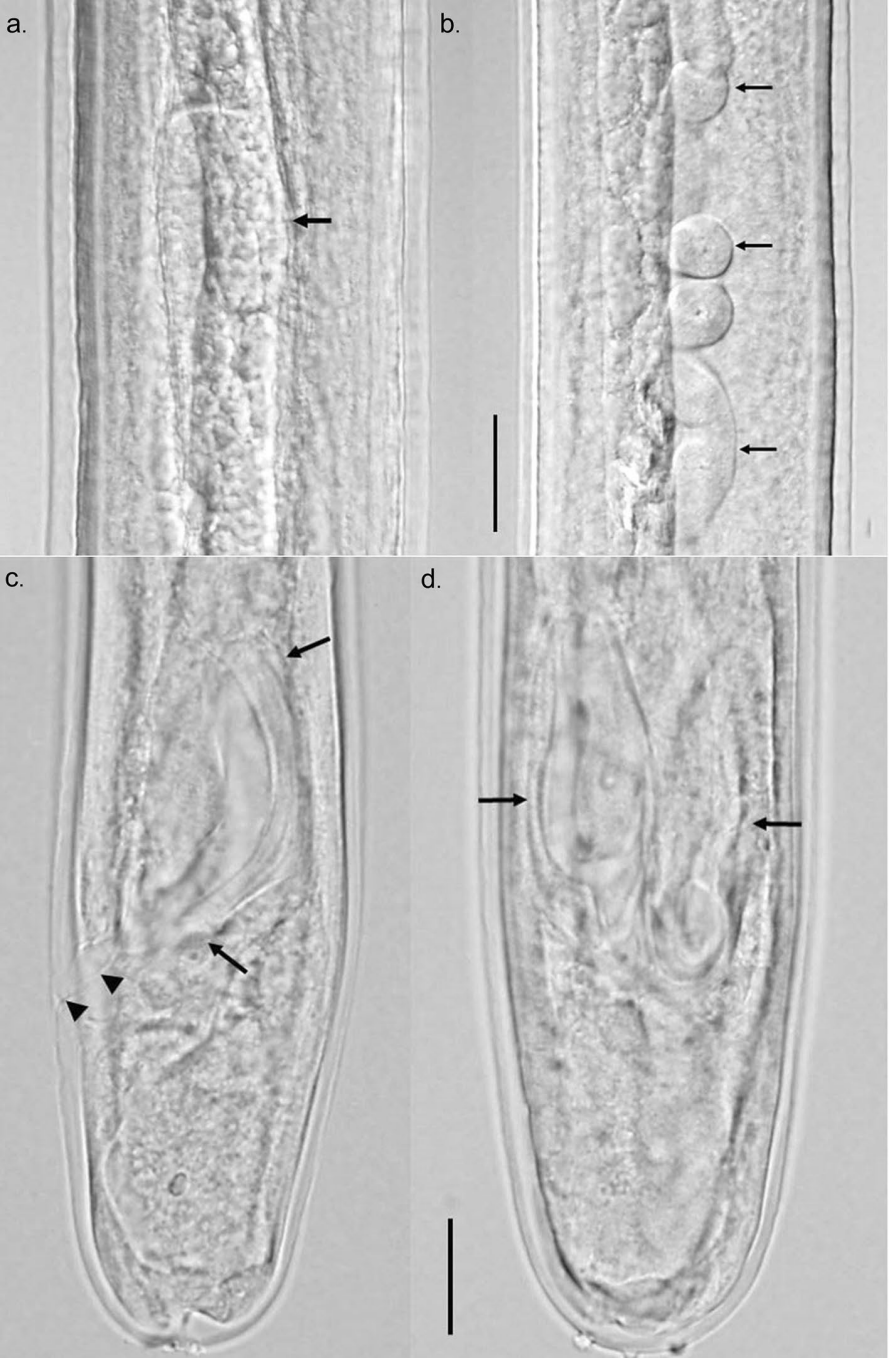
Differential Interference Contrast microscopy image of male larva recovered from NSG mice at 15 weeks post infection. Composite image of testis and reproductive tube. (**a**) C-shaped testis (arrow) at about midbody. (**b**) Highly coiled male reproductive tube (arrows) in posterior third of body. Scale bar 20 µm. Composite image of posterior end of male larva showing developing spicules. (**c**) Tail and one spicule (arrows) in lateral view showing size, shape and cuticularization. Cloaca and anal opening (arrow heads) also visible. (**d**) Dorso-ventral view showing both developing spicules (arrows). Scale bar 40 µm.

A detailed description of 15-week-old male and female larvae follows. The larvae reached an average length of approximately 7.5 mm but ranged in length from 4.4 to 10 mm. Overall, female larvae were longer than male larvae. The larvae of both sexes were long and narrow, and tapered only slightly at both ends. The esophagus was distinctly divided into anterior muscular portion and a glandular posterior portion (Fig. 2a). The ovijector was large, well formed, muscular, and with a distinct cavity, and progressed into posteriorly directed vagina vera which divided into two uterine tubes (Fig. 2c,d). The paired uterine tubes had grown to such an extent that the ovary posterior ends lay in the posterior quarter of the body (Fig. 2b). The testis, which lay at approximately mid-body, was flexed in a J-shape with the reproductive tube flexing posteriorly, highly coiled, and running the length of the body (Fig. 3a,b). The male reproductive tube joined the intestine at the posterior end of the worm to form the cloaca just ahead of the anal opening. Rudimentary developing spicules were present, measured 100–125 µm in length. They were beginning to become cuticularized but were still of same approximate size and shape (Fig. 3c,d).

### Proteomic analysis of infected NSG mice

**Figure 4:**
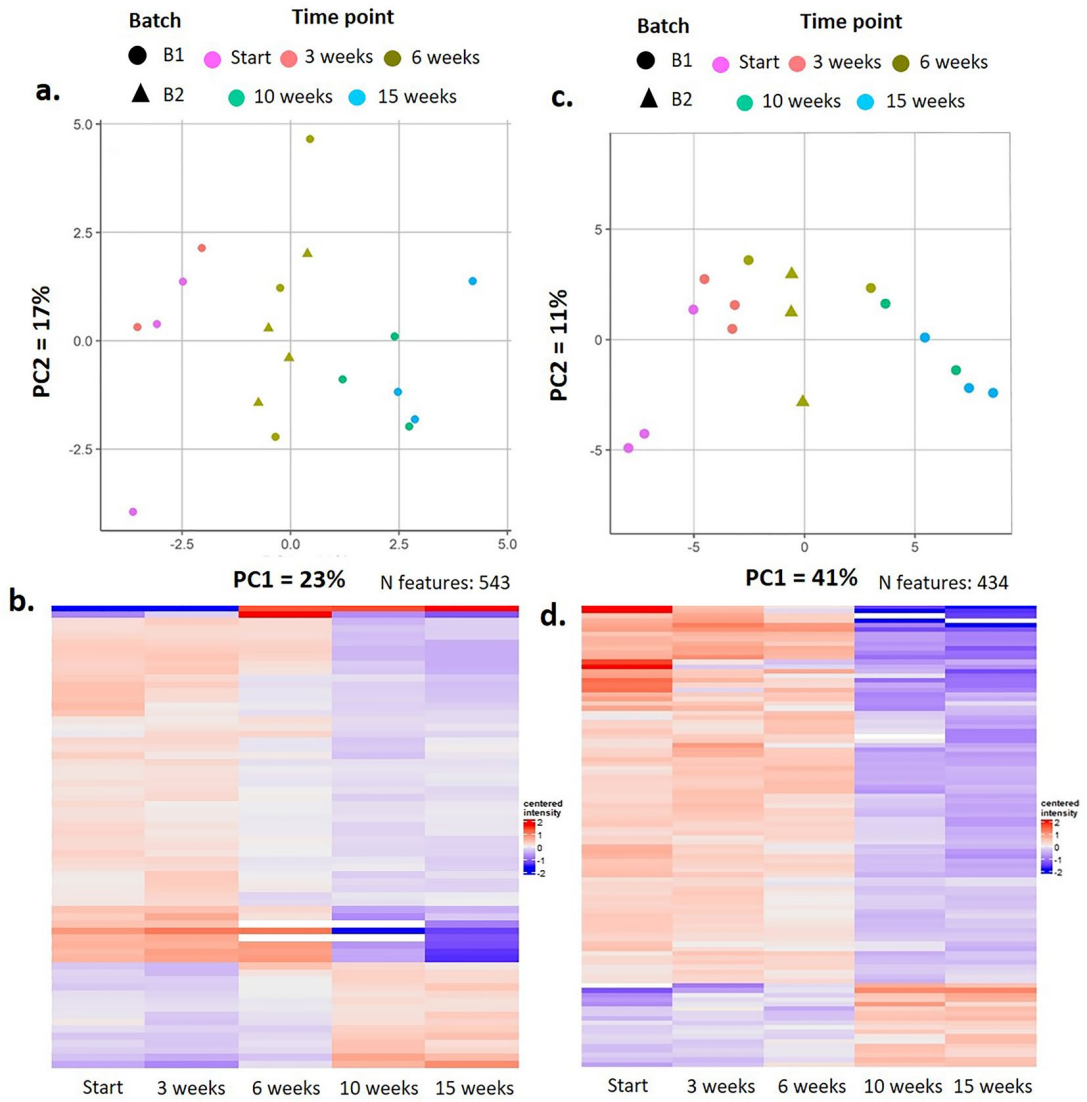
Proteomic analyses of *D. immitis* infected NSG mouse tissues. Principal component analyses (PCA) of plasma (**a**) and urine (**c**) illustrate changes in the proteome over time that are consistent within and between batches of mice and larva. Heat maps of the 28 proteins significantly altered in plasma (**b**) and 57 proteins significantly altered in urine (**d**) recapitulate the PCA analyses, displaying distinct proteomes at progressive timepoints in the infection. A general feature appears to be a downward trend in protein expression across plasma and urine as infection progresses.

Proteome composition of plasma and urine recovered from infected NSG mice was analyzed to further characterize the infection process. A total of 2712 proteins were detected, with expression profiles following various patterns in plasma and urine. Principal component analyses (PCA) illustrated a temporal sequence in proteome changes (Fig. 4a,c) that is consistent within groups and between batches of mice and larvae. Principal components are similar within the 0–3 week period yet distinct from the 6 week period, which in turn is also distinct from the 10 to 15 week period. These data may reflect the distinct stages of host manipulation that may occur as *D. immitis* larva mature and migrate into various tissues.

During the course of the infection with *D. immitis* a total of 100 proteins were significantly altered in urine and 73 in plasma (Fig. 4b,d). The observed changes included proteins organized into consistent biological categories such as downregulation of complement system components C1qc, C1ra, C2, C8a/b/, C9, and fibrinogen gamma chain. Mitochondrial proteins VDac1/2/3 displayed reduced expression in urine and plasma. Extracellular proteins such as plasminogen, Alpha-2-antiplasmin also appeared strongly reduced in urine, while collagen appeared systemically reduced in plasma. A few proteins displayed an upward trend in expression, including Annexin in urine, and Cfd and Mbl2 in plasma. Several glycosidases involved in vacuolar and lysosomal degradation (chitobiase, neuraminidase, trehalase, mannosidase 2) exhibited significant changes. Multiple major urinary proteins (MUP) were significantly reduced during the infection period, both in urine and plasma (MUP members 1, 2, 3, 17 and 20). Proteins associated with cell adhesion in epithelia varied significantly, namely cadherin-16 in plasma, and cadherin 5 in urine. Epithelial proteins, desmogleins and desmoplakin were increased in urine. Lastly, reductions in expression were observed in urine decorin and laminine, both linked to extracellular matrix, and cell adhesion (Table 1, Supplement Table 1).

**Table 1:** Proteomic analyses of *D. immitis* infected NSG mouse tissues.

Class	Molecules	Location	Action
Complement system	C1qc, C1ra, C2, C8/b/, C9	Urine and plasma	↓
	C4b	Urine	↑
Pore forming	VDac1/2/3	Urine and plasma	↓
Extracellular proteins	Plasminogen and Alpha-2-antiplasmin	Urine	↓
	Collagen	Plasma	↓
	Annexin	Urine	↑
	Cfd, Mbl2 and Fibrinogen ƴ chain	Plasma	↑
Cell adhesion (epithelia)	Cadherin-5	Urine	Varied
	Cadherin-16	Plasma	Varied
	Desmogleins and desmoplakin	Urine	↑
	Decorin and laminin	Urine	↓
Vacuolar and lysosomal activity	Chitobiase, neuraminidase, trehalase, mannosidase	Urine	↓
Multiple major urinary proteins	MUP members 1, 2, 3, 17 and 20)	Urine and plasma	↓
Renal markers	Ckb, Ckm, Ckmt1, Ckmt2, Ddah1, SDMA	Urine	Varied

Summary of proteins significantly altered in NSG mouse plasma and urine.

### Ivermectin and moxidectin are effective against *D. immitis* in NSG mice

**Figure 5:**
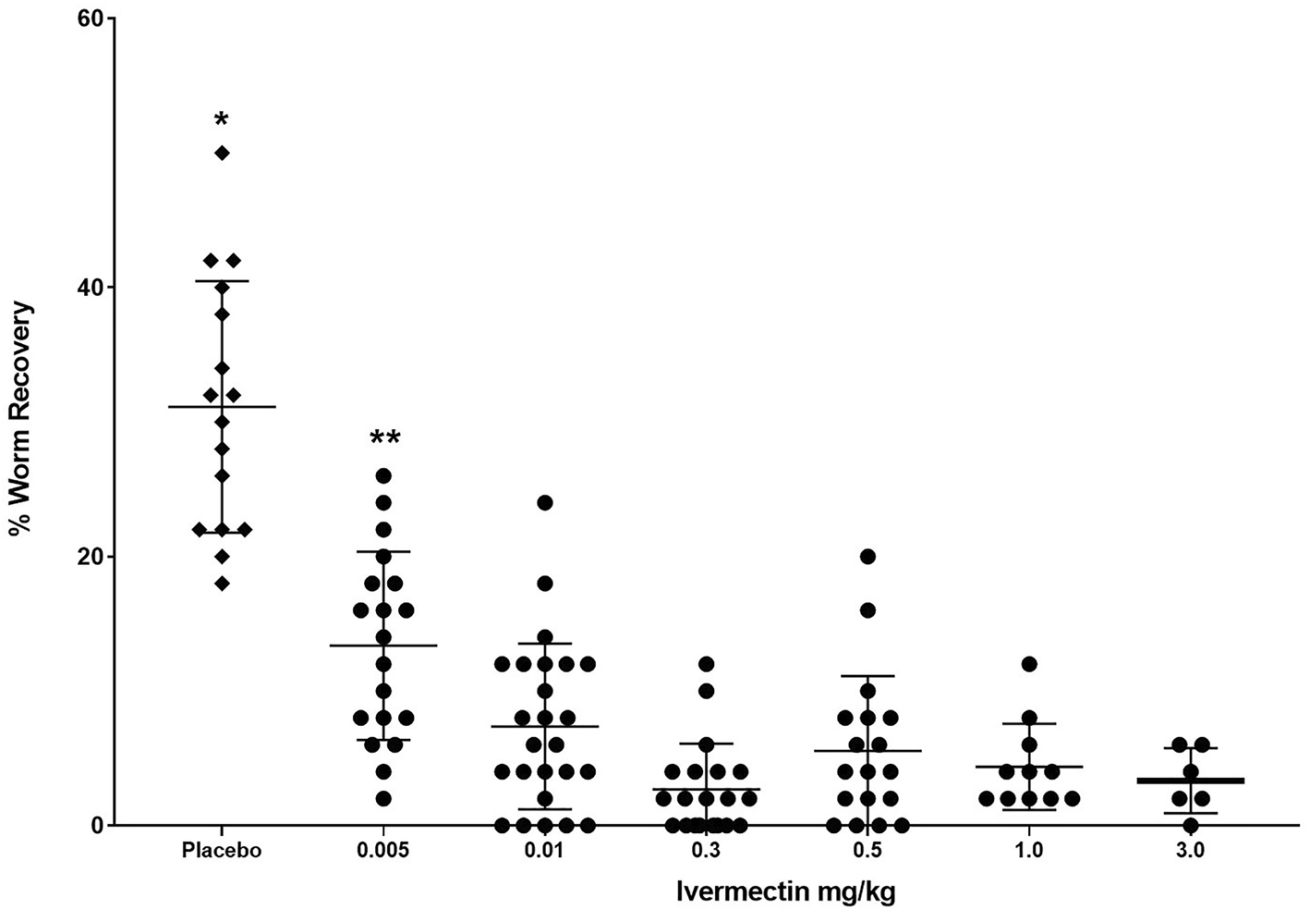
Dose–response curve of ivermectin treatment of NSG mice infected with *D. immitis*. Mice were infected for 6 weeks with the susceptible MO 2005 isolate of *D. immitis* and treated on Day 0, 15 and 30 with doses of ivermectin ranging from 0.005 to 3.0 mg/kg. *Statistically significant difference, p value ≤ 0.05 between control and ivermectin-treated groups. **Statistically significant difference, p value ≤ 0.05 between 0.005 mg/kg group recovery and parasite recovery from other ivermectin treated groups.

Mice were infected with 50 *D. immitis* L3 from the ML-susceptible MO 2005 isolate and then treated with ivermectin with a dose range of 0.005–3.0 mg/kg on days 1, 15 and 30 post-infections. Six weeks post infection, mice were necropsied and worms collected. Mice treated with 0.005 mg/kg ivermectin had a significant 57% reduction in worm survival while mice treated with 0.01 mg/kg had a significant 76% reduction in worm survival compared to controls. Treatment of mice with doses ranging from 0.3 to 3.0 mg/kg of ivermectin all had significant reductions of 87% as compared to the control group, with no differences in parasite recovery within this dose range (Fig. 5).

**Figure 6:**
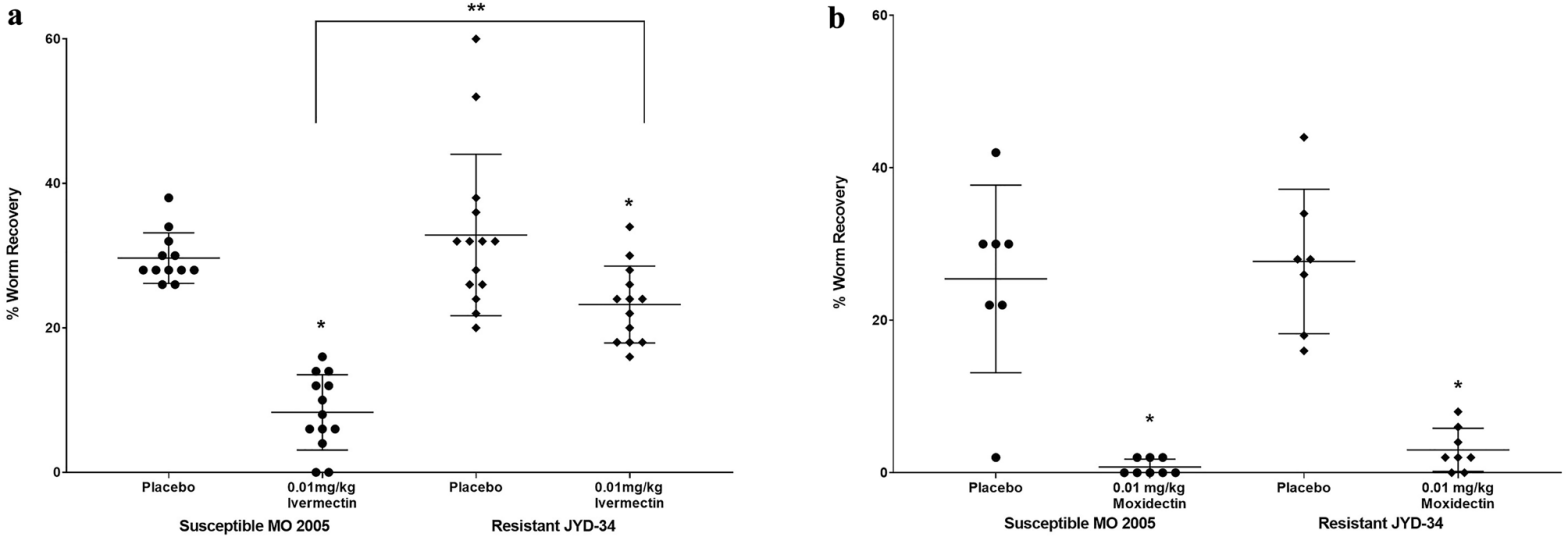
Effect of ivermectin and moxidectin on susceptible and resistant isolates of *D. immitis* in NSG mice. NSG mice were infected for 6 weeks with either the ML-susceptible MO 2005 isolate or the ML-resistant JYD-34 isolate and treated on Day 0, 15 and 30 with (**a**) 0.01 mg/kg of Ivermectin or (**b**) moxidectin. *Statistically significant difference, p value ≤ 0.05 between placebo and treated groups. **Statistically significant difference, p value ≤ 0.05 between treated susceptible and resistant isolates.

Resistance of *D. immitis* to MLs presents a significant challenge to disease prevention. To determine if a resistant phenotype can be detected in NSG mice, ML-susceptible MO 2005 isolate and the resistant JYD-34 isolate were injected into NSG mice that were then treated on days 1, 15 and 30 post-infections with 0.01 mg/kg of ivermectin or moxidectin and necropsied at six weeks post infection. Treatment with ivermectin resulted in a 73% reduction in parasite survival of the susceptible MO 2005 isolate while the resistant JYD-34 isolate had a 30% reduction compared to their relative controls. Both of these reductions in parasite survival were significantly different from their controls as well as different from each other, with the resistant isolate having a significantly higher parasite recovery as compared to the susceptible isolate (Fig. 6a). Treatment with moxidectin resulted in a 96% reduction in parasite survival of the susceptible MO 2005 while the resistant JYD-34 isolate had a statistically equivalent 88% reduction compared to their relative controls (Fig. 6b).

## Discussion

The current study demonstrated that NSG mice are susceptible to infection with *D. immitis* with larvae surviving and growing for at least 15 weeks. The fact that immunocompetent mice are resistant to the infection^10^ whereas immunodeficient NSG mice are susceptible, suggests that the innate response is capable of controlling the infection in immunocompetent mice. Previous studies in which *D. immitis* L3 were implanted in mice within diffusion chambers observed parasite survival for three weeks^11^. It is possible that the innate immune response only kills the larvae after three weeks or that the diffusion chamber provides a protective environment that affects the innate response.

Apparently, the nutritional/physiological requirements are sufficient for *D. immitis* to grow and develop in NSG mice. Infection of NSG mice with different quantities of *D. immitis* L3 resulted in the same percent survival of the parasites. This observation suggests that a percentage of the L3 are fit to survive in NSG mice and the limitation of parasite survival within the range of doses tested was not physical space in the mice. The *D. immitis* larvae were able to migrate in the mice from the site of inoculation, however none were found in the lungs or heart. Migration to the lungs/heart might have occurred by a later time point, but would likely kill the mice due to size of the worms at that point in development relative to the murine organs. Both *S. stercoralis*^16^ and *L. loa*^17^ were capable of developing into sexually mature adults in NSG mice and migrate to their appropriate niche. This was possible based on the length of the life cycle and the size of the worms. This was not possible for *O. volvulus* infections of NSG mice, in which, like *D. immitis,* development appears to be limited to development from L3 to late stage L4^18^. Regardless of this limitation, infection of humanized NSG mice with *O. volvulus* provided novel information on parasite biomarkers for the early infection in a human immune system environment^18^.

The description of *D. immitis* larval development from the L3 to the adult worm in the dog, the natural definitive host, has been well documented^8,9^. This report does not attempt to redescribe that development. Instead, this report provides a brief description of larvae recovered from NSG mice over a 15-week period, noting major similarities or differences from the published description of larval growth over that same time period in dogs. Lichtenfels et al.^8^ noted *D. immitis* larvae molting as early as 3 days in culture, whereas Orihel^9^ noted molting larvae recovered from dogs at 9 to 12 days. In the present study, larvae recovered from mice at 14 days were observed to be in the process of molting, which constitutes the 3rd overall molt and either the first molt in the vertebrate host or a second molt occurring within days of the bona fide L3–L4 molt. Lichtenfels et al.^8^ also noted the 4th and final molt to occur between 50 and 58 days in the dog, and Orihel^9^ noted that larvae had finished molting by day 70 in the dog. No molting larvae were observed between 8 and 15 weeks in the present study, therefore we cannot conclude that the larvae recovered from these mice were 5th stage larvae. It is probable that the maximum development of the larvae seen in NSG mice during the time course studied was into late 4th stage larvae. Growth of the larvae in NSG mice was equal to that described in dogs for the first four weeks. After which, larval growth accelerated in the dog while in the mouse growth rate remained constant. This change in growth dynamics might be due to space-restrictions in the mouse, absence of sufficient nutrients for optimal development, and/or lack of developmental triggers within the murine host–pathogen interactions.

A broad range of molecular changes were seen in infected NSG mice, some with strong correlations with parasite infection status. Linkage to infection with *D. immitis* for many of the proteomic changes in the infected mice remains tenuous at this time; however, there are interesting correlations between infection status and several host systems involved in control of parasitic infections. Specifically, downregulation of the coagulation and complement system components was observed in the infected NSG mice. NSG mice have been described to lack a functional complement cascade^19^ while still maintaining complement serum levels that are equivalent to those occurring in immunocompetent mice. Complement in serum from NSG mice was sufficient to collaborate in killing larval *S. stercoralis* in vitro^16^. Complement has been identified as an important factor in the control of many nematode parasites of many different stages including *D. immitis*.^20–24^. It was hypothesized that it would be beneficial for *D. immitis* to downregulate complement in NSG mice in order to promote its survival.

*Dirofilaria immitis* larvae are susceptible to elimination by ML for approximately 60 days after entering the host as an L3^4^. This window of susceptibility was convergent with the period of time that *D. immitis* survives and develops in NSG mice, thus making the model suitable for testing efficacy of ML. Ivermectin eliminated larvae in NSG mice at multiple doses. The maximum efficacy in NSG mice was 87%. It is possible that a higher efficacy would be achieved in infected NSG mice if the trials were allowed to continue beyond the 6 weeks utilized in this study. When comparing these efficacies to experimental infection of canine studies, it is important to point out that the endpoint in canines is the appearance of adults in the circulatory system, routinely assessed at 6 months post infection. Assessing the survival of larvae at early stages in mice leaves ample space for speculation concerning the translation of mouse data to the canine system. It may be the case that translation from the NSG mouse to an immunocompetent canine is dependent on drug mode of action. In the case of macrocyclic lactones, recapitulation of a resistance phenotype lends confidence that the efficacy elicited in NSG mice is truly reflective of the canine mechanism of action.

Resistance to MLs has been documented in *D. immitis*^2,5^. The mechanism of resistance is unknown. Genetic investigations in *D. immitis* and other systems have identified loci that are associated with an ML-resistant phenotype^25,26^, but a gene, or genes, that underlies resistance remains elusive. Recapitulation of a resistance phenotype has been observed in an in vitro system in which canine peripheral blood mononuclear cells and the neutrophil fraction within, adhere to *D. immitis* larvae in the presence of physiologically relevant nM concentrations of ML^27^. Importantly, an ML-resistant isolate of *D. immitis* showed diminished killing by ivermectin in NSG mice, while retaining a higher susceptibility to moxidectin^28,29^, thus demonstrating that the NSG/*D. immitis* model was suitable for evaluating compounds for efficacy against ML-susceptible and resistant isolates of *D. immitis*.

Macrocyclic lactones operate through allosteric opening of parasite glutamate gated ion channels (Glu-Cl). While multiple Glu-Cl with variable sensitivities to ML are expressed in different tissues of nematodes^30^, a target Glu-Cl has been localized to the excretory-secretory pore in *B. malayi* mf^31^. The pore is associated with neuromusculature that physically pumps molecules, collectively known as excretory-secretory products (ES), out of the nematode and into the host where they may play a role in immune evasion^32^. Treatment of *Brugia malayi* mf with ivermectin inhibits release of ES protein^31^. Additionally, it has been shown that ML inhibit the release of extracellular vesicles, which have been shown to be mediators of immune manipulation in a variety of helminth parasites^33,34^ and in *D. immitis* larvae^35^. These data suggest the MLs exert anthelmintic activity in vivo through inhibiting the parasite’s ability to manipulate the immune system. Other studies have shown that ivermectin collaborates with host cells to attach to the parasites^36^. The fact that ivermectin functions in NSG mice suggests that either the drug can function in an immune response independent mechanism or that the residual immune response in NSG mice is sufficient to collaborate with the ML to kill the larvae.

In conclusion, the present study has established that NSG mice are susceptible to infection with *D. immitis,* sustaining parasites for at least 15 weeks. *D. immitis* larvae develop into late L4 increasing in length and starting sexual maturation of both male and female worms. The host response to the infection was complex with at least some of the responses directed at reducing immune control mechanisms that remain in NSG mice. Given the short time period of efficacy of ML against *D. immitis*, the NSG model was ideal for monitoring ML activity against both susceptible and resistant isolates of parasites in vivo for screening of new chemotherapeutic agents.

## Methods and materials

### Source of parasites and mice

*Dirofilaria immitis* L3 (MO 2005 isolate) were acquired from the Filariasis Research Reagent Resource Center (FR3), Athens, GA and from Boehringer Ingelheim Animal Health, Fulton, MO (JYD-34 isolate). The MO 2005 isolate from FR3 is known to be susceptible to MLs whereas the JYD-34 isolate has been shown to have resistance to MLs in dogs^37^. *Aedes aegypti* mosquitoes were allowed to feed on microfilaremic blood^38^. After 14 days, mosquitoes were gently crushed, rinsed over a 32 µm sieve to separate the larvae, and larvae were soaked in warm RPMI, (Sigma-Aldrich, St. Louis MO.). The larvae were counted and shipped overnight maintained at ambient temperature.

C57BL/6J, NOD (NOD/ShiLt), SCID (B6.Cg-PrKdc^scid^/SzJ), NOD/SCID (NOD.Cg-Prkdc^scid^), and NSG (NOD.Cg-Prkdc^scid^Il2rg^tm1Wjl^/SzJ) mice, 6–8 weeks old, were purchased from Jackson Laboratory (Bar Harbor, ME). In addition, NSG mice were also obtained from an inhouse breeding colony. Mice were maintained at the Thomas Jefferson University animal facility and were 6–8 weeks of age at the start of an experiment. All mice were housed in the Laboratory Animal Sciences Facility in micro-isolator boxes in rooms that were specific pathogen free and under temperature, humidity and light cycle-controlled conditions. All mice were fed standard autoclavable rodent chow, except the NSG were fed a low fat 5K52 diet and given water ad libitum.

### Dirofilaria *immitis* infections and parasite recovery

L3 were washed 5 times in 1:1 mixture of NCTC-135 (Sigma-Aldrich) and Iscove’s Modified Dulbecco’s Medium (NI) (Sigma-Aldrich) with 100 U/ml penicillin plus 100 µg/ml streptomycin (Corning, Mediatech, Inc., Manassas, VA). Worms were counted and mice were infected by subcutaneous injection with a 21 g needle. Mice were infected with either 25, 50 or 100 L3 to determine the optimal dose for infection.

At 1–15 weeks post-infection, urine was collected followed by mice being sacrificed by exsanguination under isoflurane anesthesia for plasma collection. The bodies were skinned, viscera removed, and the muscles and skin were scored with blunt forceps. The body sections were soaked in RPMI media with 10% HI-FBS (Gemini Bioproducts LLC, West Sacramento, CA) and 100 U/ml penicillin plus 100 µg/ml streptomycin for 18 h at room temperature. The fluid in which the body sections were incubated was analyzed via microscopy and recovered worms were counted and fixed in warm 95% ethanol/5% glycerol and then mounted onto slides using glycerin jelly. The lengths of the recovered worms were determined using CellSens imaging software (Olympus, Center Valley, PA) and the worms were analyzed to determine stage of development using Differential Interference Contrast microscopy.

### Protein precipitation of urine samples

Proteins from each urine sample were precipitated by acetone- trichloroacetic acid (TCA) precipitation. Briefly, all samples were thawed on ice and the volume of each aliquot was determined. Samples above 250 µl were split into two aliquots. The volume of all samples and aliquots was adjusted to 175 µl with purified water. 8 volumes of ice-cold acetone (1400 µl) and 1 volume of 100% TCA with 0.4% Na-Deoxycholate (175 µl) were added to each aliquot. The samples were mixed and incubated for 60 min at − 20 °C for protein precipitation. Subsequently, samples were centrifuged at 20,000×*g* for 15 min at 4 °C and the supernatant was removed. The protein pellet was washed twice with ice-cold 80% acetone. During the second wash, the two aliquots of the split samples were pooled. Afterwards, the supernatant was completely removed, and the protein pellet was air-dried at room temperature. The protein pellet was stored at − 20 °C until further processing.

### Mass spectrometric (MS) sample preparation for mouse plasma and urine samples

The protein concentration of each plasma sample was determined with the Pierce 660 nm Protein Assay according to the manufacturer’s instructions (ThermoFisher). For MS sample preparation, the PreOmics sample preparation and peptide cleanup kit (www.preomics.com) were employed according to the vendor’s instructions^39^. Briefly, 4 µl of each mouse plasma sample and were added to 50 µl of PreOmics buffer LYSE, while the precipitated proteins of the urine samples were resuspended in 100 µl of buffer LYSE. All samples were incubated for 10 min at 95 °C for reduction and alkylation of disulfide bonds, sonicated in a water bath for three rounds of 30 s, then diluted with equal volumes (1:2) of purified water. Proteins were digested with Trypsin-LysC (Promega) overnight at 37 °C while shaking at 750 rpm. Subsequently, an equal volume of PreOmics buffer STOP was added to each sample and the treated samples were loaded onto the provided solid phase extraction cartridges. Bound peptides were consecutively washed with 2 × 200 µl of each the PreOmics wash buffers WASH-0 (urine samples only), WASH-1 and WASH-2. Purified peptides were eluted stepwise with 2 × 100 µl of PreOmics buffer ELUTE and lyophilized overnight. Purified peptides were then subjected to pre-fractionation by high pH-reversed phase chromatography^40^ for deep proteome profiling. Briefly, peptides were reconstituted in 10 mM ammonium formate (pH 10, buffer A), the peptide amount was determined by NanoDrop (ThermoFisher) measurement at 280 nm and loaded onto an XBridge C18, 200 × 4.6 mm analytical column (Waters) operated with an ÄKTA Pure system (GE Healthcare). Peptides were separated by applying a segmented gradient with increasing acetonitrile concentration from 7 to 30% buffer B (buffer A supplemented with 80% acetonitrile) over 15 min followed by a 5 min gradient to 55% buffer B. The collected fractions were combined in a concatenated way to generate a total of 6 fractions. Pooled fractions were then desalted via 100 mg SepPack C18 columns (Waters), lyophilized and reconstituted in 0.1% formic acid for subsequent MS analysis.

### Mass spectrometric analysis

All LC–MS/MS analyses were performed on a Q Exactive HF mass spectrometer (Thermo Fisher Scientific, USA) equipped with an Easy n-LC 1000 UPLC system (Thermo Fischer Scientific, USA). Samples were loaded with an auto sampler onto a 40 cm fused silica emitter (New Objective, USA) packed in-house with reversed phase material (Reprosil-Pur C_18_-AQ, 1.9 µm, Dr. Maisch GmbH, Germany) at a maximum pressure of 900 bar. The bound peptides were eluted in 120 min run time and sprayed directly into the mass spectrometer using a nano-electrospray ion source (ProxeonBiosystems, Denmark).

The Orbitrap mass spectrometer was operated in a data-dependent acquisition mode to automatically switch between full scans (resolution R = 60.000) and the acquisition of HCD fragmentation spectra (MS/MS mode) of the ten most abundant peptide ions in the Orbitrap mass analyzer (resolution R = 15.000).

### Raw data processing of samples measured in data dependent acquisition (DDA) mode

Raw files of mouse samples measured in DDA mode were processed with the MaxQuant software suite (version 1.6.0.15)^21^ for peptide and protein identification using mouse Swiss-Prot proteins with isoforms (Uniprot version 2020_03) as protein database. Carbamidomethylation of cysteine was set as a fixed modification and oxidation of methionine and N-terminal acetylation were allowed as variable modifications. The minimum required peptide length was seven amino acids and up to two missed cleavages and three labeled amino acids were allowed. A false discovery rate (FDR) of 0.01 was selected for both protein and peptide identifications and a posterior error probability (PEP) below or equal to 0.1 for each peptide-to-spectral match was required.

### Proteomics data normalization and differential expression analysis

All proteomics analyses, including data pre-processing and differential expression analysis, were conducted using R. For all downstream analyses, MaxQuant label-free quantitation (LFQ) values^41^ were log10-transformed and protein groups flagged as reverse or contaminant hits were excluded. To normalize for general loading effects between samples, a scaling factor was subtracted from the log10-transformed LFQ intensity values for each sample in all data sets. This scaling factor was calculated for each sample by subtracting the overall median of log10 LFQ values from each sample median log10 LFQ value.

To account for potential technical variability between the two sample processing batches (B1, B2), the log10-transformed LFQ intensities were adjusted for corresponding batch effects using the ‘ComBat’ function in the R package *sva* to perform a parametric batch adjustment for the known processing batch covariate separately for each tissue^42,43^. Principal component analysis (PCA) was performed on normalized and batch-corrected log10 LFQ intensities, considering only protein groups with no missing values. To test for significant protein expression differences between the ‘early’ (1 week and 3 weeks) and the ‘transition’ (6 weeks) or ‘late’ (10 weeks and 15 weeks) time points, the R package *limma*^44^ was used to fit the following linear model to the normalized and batch-adjusted log10 LFQ intensity values separately for each tissue:

Normalised intensities ~ 0 + Time point

Based on this linear regression model moderated t-tests between the ‘transition’ or ‘late’ time points and the early time point were performed for each protein group and tissue. The resulting p values were corrected for multiple hypothesis testing over all protein groups using the Benjamin-Hochberg procedure with an FDR threshold of 5%.

### Ivermectin and moxidectin treatment of NSG mice infected with *D. immitis*

Mice were infected with 50 L3 of the ML-susceptible MO 2005 FR3 isolate of *D. immitis* and then treated with ivermectin (Sigma-Aldrich Chemie GmbH, Taufkirchen, Germany) diluted in corn oil/DMSO 50:50 on days 1, 15 and 30-post infection. Each mouse was weighed on the day of treatment for individual calculation of ivermectin doses by oral gavage, ranging from 0.005 to 3.0 mg/kg. After 6 weeks, mice were sacrificed and analyzed for parasite survival as described above. NSG mice were also infected with either the ML-susceptible MO 2005 isolate or the ML-resistant JYD-34 isolate and then treated with: (1) 0.01 mg/kg of ivermectin or (2) 0.01 mg/kg moxidectin (Activate Scientific, GmbH, Prien, Germany) on days 1, 15 and 30-post infection and were sacrificed at 6 weeks post-infection and parasites recovered.

### Statistical analyses of mouse experiments

Experiments consisted of 5–7 mice per group and were performed at least twice with consistent results between experiments. Multifactorial analysis of variance ANOVA in Systat v.11 (Systat Inc., Evanstown, IL) was used to analyze the data. Probability values less than 0.05 were considered statistically significant.

### Ethical approval

All procedures and experiments in mice were performed in compliance with the ethical and regulatory standards set by the NIH for animal experimentation. Animal use protocol (01605) was approved by the Thomas Jefferson University Institutional Animal Care and Use Committee (IACUC). This protocol adhered to the “Guide for the Care and Use of Laboratory Animals” published by the National Research Council, USA. Study design, analysis, and reporting of methods and results are presented in accordance with the ARRIVE guidelines.

## Supplementary Information

Supplementary Figures.

## Data Availability

The data that support the findings of this study are available in the article and the supplementary figures and tables as well as from the corresponding author upon request.

## References

[1] Bowman DD, Atkins CE (2009). Heartworm biology, treatment, and control. Vet. Clin. N. Am. Small Anim. Pract..

[2] Noack S, Harrington J, Carithers DS, Kaminsky R, Selzer PM (2021). Heartworm disease—overview, intervention, and industry perspective. Int. J. Parasitol. Drugs Drug Resist..

[3] Simon F (2012). Human and animal dirofilariasis: the emergence of a zoonotic mosaic. Clin. Microbiol. Rev..

[4] Bowman DD, Drake J (2017). Examination of the “susceptibility gap” in the treatment of canine heartworm infection. Parasit. Vectors.

[5] Prichard RK (2021). Macrocyclic lactone resistance in *Dirofilaria immitis*: risks for prevention of heartworm disease. Int. J. Parasitol..

[6] Selzer PM, Epe C (2021). Antiparasitics in animal health: Quo Vadis?. Trends Parasitol..

[7] Kotani T, Powers KG (1982). Developmental stages of *Dirofilaria immitis* in the dog. Am. J. Vet. Res..

[8] Lichtenfels J, Pilitt P, Kotani L, Powers K (1985). Morphogenesis of development stages of *Dirofilaria immitis* (Nematoda) in the dog. Proc. Helminthol. Soc. Wash..

[9] Orihel TC (1961). Morphology of the larval stages of *Dirofilaria immitis* in the dog. J. Parasitol..

[10] Delves CJ, Howells RE (1985). Development of *Dirofilaria immitis* third stage larvae (Nematoda: Filarioidea) in micropore chambers implanted into surrogate hosts. Trop. Med. Parasitol..

[11] Abraham D, Grieve RB, Mika-Grieve M, Seibert BP (1988). Active and passive immunization of mice against larval *Dirofilaria immitis*. J. Parasitol..

[12] Shultz LD (2005). Human lymphoid and myeloid cell development in NOD/LtSz-scid IL2R gamma null mice engrafted with mobilized human hemopoietic stem cells. J. Immunol..

[13] Bosma MJ, Carroll AM (1991). The SCID mouse mutant: definition, characterization, and potential uses. Annu. Rev. Immunol..

[14] Serreze DV, Gaskins HR, Leiter EH (1993). Defects in the differentiation and function of antigen presenting cells in NOD/Lt mice. J. Immunol..

[15] Shultz LD (1995). Multiple defects in innate and adaptive immunologic function in NOD/LtSz-scid mice. J. Immunol..

[16] Patton JB (2018). Methylprednisolone acetate induces, and Delta7-dafachronic acid suppresses, *Strongyloides stercoralis* hyperinfection in NSG mice. Proc. Natl. Acad. Sci. USA.

[17] Pionnier NP (2019). Mouse models of Loa loa. Nat. Commun..

[18] Patton JB (2018). Development of *Onchocerca volvulus* in humanized NSG mice and detection of parasite biomarkers in urine and serum. PLoS Negl. Trop. Dis..

[19] Brehm MA, Wiles MV, Greiner DL, Shultz LD (2014). Generation of improved humanized mouse models for human infectious diseases. J. Immunol. Methods.

[20] El-Sadr WM, Aikawa M, Greene BM (1983). In vitro immune mechanisms associated with clearance of microfilariae of *Dirofilaria immitis*. J. Immunol..

[21] Garza JJ, Greiner SP, Bowdridge SA (2017). Serum-mediated *Haemonchus contortus* larval aggregation differs by larval stage and is enhanced by complement. Parasite Immunol..

[22] Giacomin PR (2008). The role of complement in innate, adaptive and eosinophil-dependent immunity to the nematode *Nippostrongylus brasiliensis*. Mol. Immunol..

[23] Chandrashekar R, Rao UR, Parab PB, Subrahmanyam D (1986). *Brugia malayi*: rat cell interactions with infective larvae mediated by complement. Exp. Parasitol..

[24] Kerepesi LA, Hess JA, Nolan TJ, Schad GA, Abraham D (2006). Complement component C3 is required for protective innate and adaptive immunity to larval *Strongyloides stercoralis* in mice. J. Immunol..

[25] Bourguinat C (2015). Macrocyclic lactone resistance in *Dirofilaria immitis*: Failure of heartworm preventives and investigation of genetic markers for resistance. Vet. Parasitol..

[26] Evans KS (2021). Two novel loci underlie natural differences in *Caenorhabditis elegans* abamectin responses. PLoS Pathog..

[27] Berrafato T, Coates R, Reaves BJ, Kulke D, Wolstenholme AJ (2019). Macrocyclic lactone anthelmintic-induced leukocyte binding to *Dirofilaria immitis* microfilariae: Influence of the drug resistance status of the parasite. Int. J. Parasitol. Drugs Drug Resist..

[28] McTier TL (2021). Comparative preventive efficacy of ProHeart((R)) 12, Heartgard((R)) Plus and Interceptor((R)) Plus against a macrocyclic lactone-resistant strain (JYD-34) of heartworm (*Dirofilaria immitis*) in dogs. Parasit. Vectors.

[29] Savadelis MD, McTier TL, Kryda K, Maeder SJ, Woods DJ (2022). Moxidectin: heartworm disease prevention in dogs in the face of emerging macrocyclic lactone resistance. Parasit. Vectors.

[30] Wolstenholme AJ (2012). Glutamate-gated chloride channels. J. Biol. Chem..

[31] Moreno Y, Nabhan JF, Solomon J, Mackenzie CD, Geary TG (2010). Ivermectin disrupts the function of the excretory-secretory apparatus in microfilariae of *Brugia malayi*. Proc. Natl. Acad. Sci. USA.

[32] Moreno Y, Geary TG, Tritten L (2021). When secretomes meet anthelmintics: lessons for therapeutic interventions. Trends Parasitol..

[33] Tritten L, Geary TG (2018). Helminth extracellular vesicles in host–parasite interactions. Curr. Opin. Microbiol..

[34] Hoffmann KF, Hokke CH, Loukas A, Buck AH (2020). Helminth extracellular vesicles: great balls of wonder. Int. J. Parasitol..

[35] Harischandra H, Yuan W, Loghry HJ, Zamanian M, Kimber MJ (2018). Profiling extracellular vesicle release by the filarial nematode *Brugia malayi* reveals sex-specific differences in cargo and a sensitivity to ivermectin. PLoS Negl. Trop. Dis..

[36] Vatta AF (2014). Ivermectin-dependent attachment of neutrophils and peripheral blood mononuclear cells to *Dirofilaria immitis* microfilariae in vitro. Vet. Parasitol..

[37] Bowman DD (2017). Evaluation of the efficacy of ProHeart((R)) 6 (moxidectin) against a resistant isolate of *Dirofilaria immitis* (JYD-34) in dogs. Parasit. Vectors.

[38] McCall J (1981). The role of arthropods in the development of animal models for filariasis research. J. Georgia Entomol. Soc..

[39] Kulak NA, Pichler G, Paron I, Nagaraj N, Mann M (2014). Minimal, encapsulated proteomic-sample processing applied to copy-number estimation in eukaryotic cells. Nat. Methods.

[40] Wang Y (2011). Reversed-phase chromatography with multiple fraction concatenation strategy for proteome profiling of human MCF10A cells. Proteomics.

[41] Cox J (2014). Accurate proteome-wide label-free quantification by delayed normalization and maximal peptide ratio extraction, termed MaxLFQ. Mol. Cell Proteom..

[42] Leek, J. T., *et al.**sva: Surrogate Variable Analysis. R package version 3.46.0.* http://www.bioconductor.org/packages/release/bioc/html/sva.html (2022).

[43] Johnson WE, Li C, Rabinovic A (2007). Adjusting batch effects in microarray expression data using empirical Bayes methods. Biostatistics.

[44] Ritchie ME (2015). limma powers differential expression analyses for RNA-sequencing and microarray studies. Nucleic Acids Res..

